# Interaction between depressive symptoms and obesity-related anthropometric measures on multimorbidity among community-dwelling older adults: evidence from India

**DOI:** 10.1186/s12889-024-17894-3

**Published:** 2024-02-07

**Authors:** Waquar Ahmed, T. Muhammad, CV Irshad

**Affiliations:** 1 Department of Health Systems Studies, Tata Institute of Social Sciences, Mumbai, India; 2 Pennsylvania State University, University Park, USA; 3 School of Social Sciences and Languages, Vellore Institute of Technology, Vellore, India

**Keywords:** Depressive symptoms, Body composition, Multimorbidity, Older adults

## Abstract

**Background:**

This study aimed to examine the associations between depressive symptoms, body mass index (BMI), waist circumference, waist-hip ratio and multimorbidity among community-dwelling older adults. We also examine the interaction effects between depressive symptoms, BMI, waist circumference and waist-hip ratio on multimorbidity among older adults in India.

**Methods:**

A cross-sectional study was conducted, and the data were obtained from the Longitudinal Ageing Study in India (LASI) wave-1, with a sample of 31,464 older adults aged 60 years and above (men-15,098 and women-16,366). We used multinomial logistic regression to explore the independent associations between depressive symptoms, obesity-measures, and single and multimorbidity. We also estimated the interaction effects of depressive symptoms and obesity-measures on multimorbidity.

**Results:**

The prevalence of multimorbidity was higher among individuals with depressive symptoms (39.22%) than individuals with no depressive symptoms (29.94%). Adjusted models indicated that older adults with depressive symptoms had higher odds of single and multimorbidity [(AOR = 1.40, 95% CI: 1.17–1.68) and (AOR = 1.85, 95% CI: 1.58–2.16), respectively]. Similarly, in comparison to the normal BMI category, overweight and obese older adults were more likely to report single morbidity [(AOR = 1.62, 95% CI: 1.37–1.92 and (AOR = 2.14, 95% CI: 1.67–2.75), respectively] and multimorbidity [(AOR = 2.00, 95% CI: 1.72–2.33) and (AOR = 3.77, 95% CI: 2.94–4.82), respectively].

**Conclusion:**

The findings revealed that the presence of depressive symptoms, overweight or obesity, and high-risk anthropometric measures such as high-risk waist circumference and high-risk waist to hip ratio significantly increased the risk of morbidity among older adults in India. Thus, it is suggested to adopt an integrated public health policy approach to control depressive symptoms and high-risk body composition to strategically prepare against the elevated risk of multimorbidity among ageing populations.

## Background

 Globally, 5.7% of adults older than 60 years have depression, which is a leading cause of disability in the world and significantly contributes to the burden of disease [1]. In India, 45.7 million of the population had depressive disorder [2], and the prevalence of depressive disorder was observed to be increasing during adulthood and old age, which has significant implications due to the rapid ageing of the Indian population [2].

Studies reported that there is a strong negative impact of late life depression on the somatic multimorbidity condition [3]. It was also found that depressive symptoms and other poor mental health conditions are associated with chronic conditions such as hypertension [4, 5]. Prevalence of depressive symptoms with chronic conditions may further lead to severe health consequences. A large-scale sample based longitudinal study conducted among ageing population indicated that the combined effect of depressive symptoms and type-2 diabetes increased the risk of adverse health outcomes and mortality [6].

According to the world health organization (WHO), more than 650 million people were obese in 2016 and obesity has increased three times worldwide since 1975 [7]. The leading cause of high body mass index (BMI) related disability adjusted life years (DALYs) is cardiovascular disease, and together with diabetes, kidney disease and neoplasm accounted for 89.3% of all high BMI-related DALYs [8]. One study reported that individuals who were overweight or obese had two times the risk of having comorbid chronic conditions such as hypertension, osteoarthritis, dyslipidemia and diabetes [9]. According to one multiple cohort study, there was an association between obesity and 21 diseases (cardiometabolic, digestive, respiratory, neurological, musculoskeletal, and infectious diseases), which were strongly connected such that one disease increased the risk of development of another, and association with many diseases were found to be bidirectional. The most prevalent obesity-related disease in individuals with obesity and complex multimorbidity were diabetes, hypertension, sleep disorders, osteoarthritis, arrhythmias, bacterial infections and asthma [10].

Comorbid depression and obesity among older adults have a relationship with the increased risk of non-communicable diseases. According to a recent study, in comparison to the participants without comorbid depression and obesity, those having comorbid depression and obesity had 6.7 times increased risk of diabetes and 7.6 times increased risk of hypertension [11]. According to a meta-analysis, there were bidirectional associations between depression and obesity. The study reported that those suffering from depression had a 37% higher risk of being obese than those without depression, and obese people had an 18% higher risk of suffering from depression [12]. This strength of the relationship is higher from obesity to depression [13]. Additionally, obesity affects mental and cognitive health, as it is associated with an increased risk of depression and dementia [14, 15]. Obesity may also affect how individuals feel about themselves and what people living in society feel about them. Obesity is generally associated with the perception of lower level of self-esteem [16].

Besides, several factors including genetic, psychosocial, socioeconomic and behavioral ones may affect the relationship between depression, obesity and multimorbidity. Family history is one of the strong non-modifiable risk factors for coronary heart disease [17], stroke [18], diabetes [19], hypertension [20], breast cancer, lung cancer, colorectal cancer, prostate cancer, and ovarian cancer [21]. Similarly, socioeconomic status, place of residence and dietary pattern are associated with the increased risk of cardiovascular diseases among older adults [22–25]. Moreover, experiencing elder abuse [26, 27], adverse lifestyle behaviors such as increased alcohol use [28, 29] and tobacco consumption [30, 31] are associated with higher rates of chronic diseases among individuals.

**Fig. 1:**
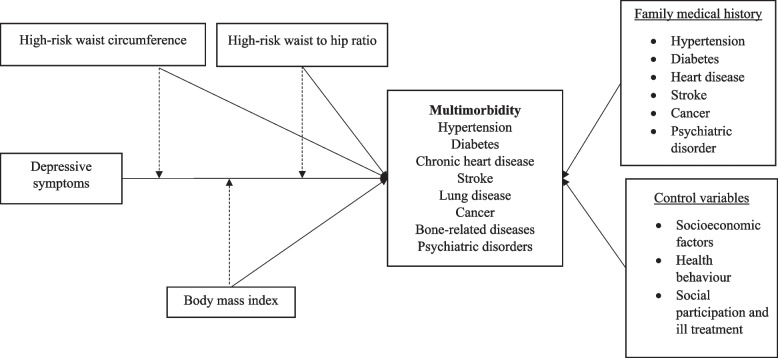
Conceptual framework of the study

Existing evidence suggests a combined effect of obesity, abdominal obesity and psychological disorders such as depression and anxiety on physical quality of life [32]. Therefore, understanding the independent associations between depressive symptoms, obesity-related biomarkers and multimorbidity can help identify the vulnerable subpopulation groups in terms of framing programmatic interventions in reducing chronic disease risk among older population. Based on the conceptual framework summarized in Fig. 1, we aimed to examine the associations between depressive symptoms, BMI, waist circumference, waist-hip ratio and multimorbidity among community-dwelling older adults. We also examine the interaction effects between depressive symptoms, BMI, waist circumference and waist-hip ratio on multimorbidity among older adults in India, after adjusting for potential confounders.

## Methods

### Study design and sample

**Fig. 2:**
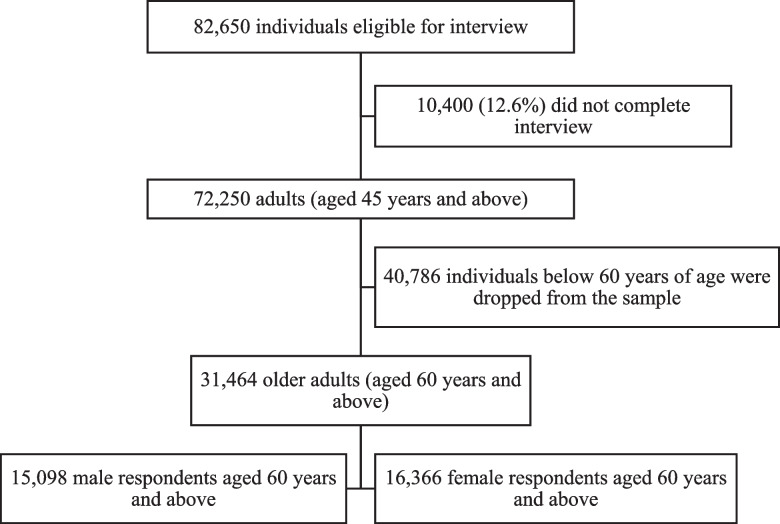
Sample selection procedure

A cross-sectional study design was used, and the data were taken from the Longitudinal Ageing Study in India (LASI) wave-1, which was collected between 2017 and 2019 [33]. The LASI is a nationally representative survey that included 72,250 individuals aged 45 and above along with their spouses (irrespective of age) in India’s states and union territories. The goal of the study was to gather information on the socioeconomic and health status of older persons in India. The present study is conducted on eligible older adults 60 years of age and above in India. The overall sample consisted of 31,464 older adults (men-15,098 and women-16,366) (Fig. 2).

#### Procedure

The LASI survey used a three-stage sample strategy and a four-stage sampling design in rural and urban areas. Primary sampling units (PSUs), that is, subdistricts (Tehsils/Talukas), were selected in each state and union territory (UT) in the first stage, while villages and wards in rural and urban areas were selected respectively in the second stage. In the third stage, households in selected villages were selected in rural areas. However, sampling in urban areas required an additional stage. In the third stage, one census enumeration block (CEB) was specifically selected randomly in each urban area. From this CEB, households were selected for the fourth stage. In each stage of sample selection, the main aim was to select a representative sample. In addition, each consenting respondent who was 45 years of age or older and their spouses (regardless of age) in the sample household received an individual survey schedule. Furthermore, the LASI also has a separate module on direct health examination and biomarkers. The survey report includes a detailed methodology and complete information about the survey design and data collection [33]. The survey agencies obtained the respondents’ prior consent before conducting the field survey for the data collection.

#### Ethics statement

The study was approved by the “Indian Council of Medical Research (ICMR) Ethics Committee in January 2017 and written or oral informed consent was obtained from the participants.” All methods were carried out in accordance with relevant guidelines and regulations and in accordance with the World Medical Association Declaration of Helsinki.

#### Outcome variable

Multimorbidity (no/single/multiple) was the outcome variable. When two or more chronic diseases coexist, this is referred to as having a multimorbidity condition and the chronic conditions in this study include hypertension, diabetes, cancer, chronic heart diseases, stroke, chronic lung disease, bone/joint disease, and neurological/psychiatric disease. Each disease was assessed using the survey question, ‘Has any health professional ever diagnosed you with the following chronic conditions or diseases?’. The responses were coded as no and yes for each chronic disease and therefore considered as self-reported.

##### Key explanatory variable

The key independent variable in this study was ‘depressive symptoms’. Depressive symptoms was assessed using the CIDI-SF (Short Form Composite International Diagnostic Interview) scale [34, 35]. It has three screening questions (based on the presence of dysphoria and/or anhedonia for ≥ 2 weeks during the past 12 months) and seven symptom-based questions. The seven symptoms include loss of interest, feeling tired, loss of appetite, trouble concentrating, feeling of worthlessness, thinking about death and trouble falling asleep [36]. A positive answer to three or more of those lead to the attribution of the label “diagnosed with depressive symptoms”. This scale estimates a probable psychiatric diagnosis of depressive symptoms and has been validated in field settings [36]. The scale was validated with well-established cross-cultural applicability especially by non-clinicians in general population surveys and widely used in population-based health surveys [36–38]. Cronbach’s alpha indicated that CIDI-SF has an acceptable reliability (α = 0.68).

Other key explanatory variables include older adults’ high-risk waist-hip ratio, high-risk waist circumference, and overall overweight/obesity status. These body measurements were obtained following standardised protocols by qualified experts. Participants were instructed to stand straight up against a wall with their bare feet on the stadiometer base to measure height. Participants were dressed in light clothing without footwear and weighed while standing on a scale. The BMI was computed by dividing the weight (in kilogrammes) by the square of height (in meters). The respondents were categorised as underweight, normal, overweight and obese. BMI was recoded as underweight (less than 18.5 ${kg/m}^{2}$), normal (18.5 to 24.9 ${kg/m}^{2}$), overweight (25-29.9 ${kg/m}^{2}$) and obese (30 ${kg/m}^{2}$ and above) [39].

While wearing light clothing and standing, the participant’s waist and hip circumference were measured using a soft gulik tape. High-risk waist circumference was defined as a male or female having a waist circumference greater than 102 cm or 88 cm, respectively. High-risk waist-hip ratio was identified when waist-hip ratios for males and females were more than or equal to 0.90 and 0.85, respectively. Finally, information on family medical history of chronic conditions was collected using the question “We would like to know about the medical history of your family. Could you tell me if your father, mother, brother, sister, children, grandchildren, has ever been diagnosed with the following diseases? Please only refer to blood-related family members”. Reporting a history of disease among any family member was considered for the explanatory variables of family medical history for all of the chronic conditions mentioned above. 

##### Control variables

In addition, socioeconomic, demographic, and behavioural factors were considered as control variables in this study. Age was categorised into young old (60–69 years), old–old (70–79 years), and oldest-old (80 + years). Education was coded as no, primary, secondary, and higher. Marital status was coded as currently married, widowed, and others (separated/ never married/divorced). Working status was coded as never worked, working, retired, and not working. Smoking tobacco and chewing tobacco were coded as no and yes. Physical activity status was coded as no, only moderate, only vigorous, and both moderate and vigorous. Alcohol drinking was coded as no and yes. Social participation was coded as no and yes, and ill-treated was coded as no and yes. The family history of hypertension, diabetes, chronic heart disease, stroke, cancer and psychiatric disease was measured using the question “We would like to know about the medical history of your family. Could you tell me if your father, mother, brother, sister, children, grandchildren, has ever been diagnosed with the following diseases? Please only refer to blood-related family members”. If the individual reported the history of disease among any of the above-mentioned family members, they were considered as having family history of respective disease and otherwise, no.

The monthly per capita expenditure quintile (MPCE) was evaluated using household consumption data. MPCE was categorised into five quintiles, i.e., from poorest to richest. Religion was coded as Hindu, Muslim, Christian, and Others. The social group (caste) was coded as Scheduled Tribes, Scheduled Castes, Other Backward Classes, and others. The place of residence was coded as rural and urban. The region was coded as North, Central, East, Northeast, West, and South.

#### Statistical approach

Descriptive analysis was conducted to report the percentage distribution of the sample across background variables. Cross-tabulations were conducted to examine the prevalence of single and multimorbidity among older participants by depression and obesity-related measures. Further, multinomial logistic regression has been used to explore the independent associations between depressive symptoms, obesity-measures and, single and multimorbidity.

Multinomial logistic regression equation is written as:

$$OR=\,\frac{P\left(Y=1|X+1\right)/\,P(Y=base\,category|X+1)}{P\left(Y=1|X\right)/\,P(Y=base\,category|X)}$$

The estimates of multinomial logistic regression are presented in the form of crude and adjusted odds ratio (OR) and P is the probability of occurrences in the equation. The crude OR represents the estimates of the association of a specific predictor variable with the outcome variable while adjusted OR represents the association of respective variables with the outcome variable despite adjustment for other control variables in the model. Multinomial logistic regression was used with three categories of multimorbidity: (i) no morbidity, (ii) single morbidity and (iii) multimorbidity. No morbidity was taken as base category and first category of independent variables was taken as the base category. In addition, we have estimated the interaction effects of depressive symptoms and obesity-related anthropometric measures (body mass index, high-risk waist circumference, and high-risk waist to hip ratio) on multimorbidity by stratifying the population into groups in the regression model.

Individual survey weights were used to account for the complex survey design and to make the estimates nationally representative. Regression diagnostics, such as variance inflation factor (VIF) for testing multicollinearity, were carried out and found no violation of basic assumptions of regression. Further, in this study, there is a potential for endogeneity in the model due to possible bidirectional relationship between depression (exposure) and multimorbidity (outcome). To address this, we looked for possible instrumental variable. We considered “family history of psychiatric disorders” as a potential instrumental variable. This instrumental variable was assessed based on whether any blood related family member of older adults was ever diagnosed by any psychiatric disorders.

Since our study outcome was categorical, we employed instrumental variable probit model (“ivprobit” in Stata) to confirm the problem of endogeneity. However, the analysis with instrumental variable indicated that there is no endogeneity problem. We arrived at this conclusion based on the Wald test of exogeneity employed in the instrumental variable probit model. It was found that the Wald test of exogeneity statistic [*p* value = 0.225] was not significant and thereby, we accepted the null hypothesis that “there is no endogeneity”. Therefore, using standard logit model was acceptable. All the analysis was carried out in STATA-15 software.

## Results

 Table 1 presents sample characteristics of the study variables. The weighted column percentage indicates representative sample characteristics adjusted for the potential sampling concerns such as over-representation or under-representation. Mean age of the study sample was 68.88 (Standard deviation: 7.51). A majority of the study participants age 60–69 years (58.51%) and were females (52.55%). More than half of the study sample had no formal schooling (56.52%), whereas only 7.74% of older adults earned higher education. 61.63% of the older adults were in a marital union, and 36.20% were widowed. More than a quarter of the study participants never worked during their lifetime (26.43%), and 29.87% were currently working. A high number of older adults were not engaged in physical activity (62.41%), whereas a little over a quarter of the study participants were engaged in vigorous physical activity (26.44%). It was found that relatively only a lower proportion of the study sample was exposed to health risk behaviours such as alcohol drinking (5.35%), smoking tobacco (15.56%), and chewing tobacco (24.62%). Most of the study participants maintained some form of social participation (53.17%). 5.22% of the study participants were exposed to ill treatment. More representation of the study participants was constituted by Hindus (82.22%), OBC caste category (45.23%), and rural residents (70.550%).

**Table 1:** Sample characteristics, Longitudinal Aging Study in India, 2017-19

Variables	Categories	N	w col %
**Age**	60–69	18,974	58.51
	70–79	9,101	30.20
	80+	3,389	11.29
**Sex**	Male	15,098	47.45
	Female	16,366	52.55
**Education**	No	16,889	56.52
	Primary	5,840	17.50
	Secondary	6,106	18.24
	Higher	2,629	7.74
**Marital status**	Currently in union	19,920	61.63
	Widowed	10,719	36.20
	Others	825	2.17
**Work status**	Never worked	8,784	26.43
	Not working	10,990	36.45
	Working	8,997	29.87
	Retired	2,693	7.25
**Physical activity**	No	19,864	62.41
	Moderate only	1,645	5.19
	Vigorous only	8,314	26.44
	Moderate and vigorous	1,166	4.06
**Alcohol drinking**	No	29,163	94.65
	Yes	2,044	5.35
**Smoke tobacco**	No	26,019	84.44
	Yes	5,185	15.56
**Chew tobacco**	No	24,211	75.38
	Yes	6,993	24.62
**Social participation**	No	13,792	46.83
	Yes	17,223	53.17
**Ill-treated**	No	29,157	94.78
	Yes	1,270	5.22
**MPCE quintile**	Poorest	6,484	21.70
	Poorer	6,477	21.71
	Middle	6,416	20.95
	Richer	6,170	19.19
	Richest	5,917	16.45
**Religion**	Hindu	23,037	82.22
	Muslim	3,731	11.28
	Christian	3,150	2.86
	Others	1,546	3.64
**Social group**	SC	5,140	18.91
	ST	5,173	8.12
	OBC	11,886	45.23
	Others	9,265	27.74
**Residential status**	Urban	10,739	29.45
	Rural	20,725	70.55
**Country regions**	North	5,812	12.59
	Central	4,262	20.95
	East	5,757	23.64
	Northeast	3,752	2.97
	South	7,578	22.68
	West	4,303	17.17

*N* Un-weighted counts, *w col %* Column percentages weighted to account for the complex survey design and to make the estimates nationally representative, *MPCE* Monthly per capita consumption expenditure, *SC* Scheduled caste, *ST* Scheduled tribe, *OBC* Other backward classes

 Table 2 presents the percentage prevalence of morbidity by key predictor variables among older adults. Overall, 8.67% of older adults reported depressive symptoms whereas, 26.89% were underweight and 5.53% were obese in this study. A total of 23.44% had a high-risk waist circumference and 77.04% had a high-risk waist to hip ratio. Among those with no depressive symptoms, 48.78% had no morbidity, whereas 39.61% of older adults with depressive symptoms had no morbidity condition. The prevalence of multimorbidity was higher among individuals with depressive symptoms (39.22%) than individuals with no depressive symptoms (29.94%). Similarly, the prevalence of multimorbidity was higher among overweight (44.88%) and obese (58.06%) older adults compared to underweighted (19.04%) and normal (28.98%) older adults. More than half of the underweight (61.9%) and normal (50.34%) older adults did not report any morbidity. The results further revealed that 72.02% of older adults had multimorbidity if they suffered from depressive symptoms and obesity.

**Table 2:** Single and multiple morbidity prevalence across categories of key predictor variables, Longitudinal Aging Study in India, 2017-19

Variables	Categories	No morbidity	Single morbidity	Multimorbidity	Total sample
		N (row %)	N (row %)	N (row %)	N (column %)
**Depressive symptoms**	No	13,654 (48.78)	5958 (21.28)	8381 (29.94)	27,992 (91.33)
	Yes	1052 (39.61)	562 (21.17)	1042 (39.22)	2,657 (8.67)
**BMI**	Normal	7138 (50.34)	2933 (20.68)	4110 (28.98)	14,181 (51.02)
	Underweight	4628 (61.9)	1425 (19.06)	1424 (19.04)	7,476 (26.89)
	Overweight	1392 (30.22)	1147 (24.91)	2067 (44.88)	4,606 (16.57)
	Obese	289 (18.82)	355 (23.12)	892 (58.06)	1,537 (5.53)
**High-risk waist circumference**	No	11,726 (54.61)	4315 (20.1)	5430 (25.29)	21,470 (76.56)
	Yes	1795 (27.3)	1603 (24.38)	3178 (48.33)	6,575 (23.44)
**High-risk waist to hip ratio**	No	3741 (58.16)	1197 (18.6)	1494 (23.23)	6,432 (22.96)
	Yes	9774 (45.27)	4719 (21.86)	7097 (32.87)	21,590 (77.04)
**Depressive symptoms & BMI**	No & Normal	6665 (51.33)	2685 (20.67)	3636 (28)	12,986 (46.77)
	No & Underweight	4216 (62.81)	1273 (18.96)	1224 (18.23)	6,712 (24.17)
	No & Overweight	1298 (30.39)	1068 (25)	1906 (44.61)	4,272 (15.38)
	No & Obese	278 (19.27)	340 (23.57)	824 (57.16)	1,442 (5.19)
	Yes & Underweight	461 (39.14)	247 (20.96)	470 (39.9)	1,178 (4.24)
	Yes & Normal	408 (53.88)	151 (19.94)	198 (26.18)	758 (2.73)
	Yes & Overweight	92 (27.96)	79 (23.91)	158 (48.13)	329 (1.19)
	Yes & Obese	11 (11.95)	15 (16.04)	67 (72.02)	94 (0.34)
**Depressive symptoms & High-risk waist circumference**	No & No	10,882 (55.56)	3926 (20.05)	4778 (24.4)	19,586 (69.92)
	No & Yes	1648 (27.24)	1493 (24.68)	2908 (48.07)	6,049 (21.59)
	Yes & No	827 (44.53)	385 (20.72)	646 (34.75)	1,858 (6.63)
	Yes & Yes	146 (27.98)	109 (20.87)	266 (51.15)	521 (1.86)
**Depressive symptoms & High-risk waist to hip ratio**	No & No	3440 (59.28)	1062 (18.3)	1301 (22.42)	5,802 (20.73)
	No & Yes	9086 (45.86)	4356 (21.98)	6372 (32.16)	19,814 (70.78)
	Yes & No	298 (47.8)	134 (21.46)	192 (30.73)	624 (2.23)
	Yes & Yes	674 (38.45)	360 (20.54)	718 (41.01)	1,752 (6.26)
**Family medical history**					
** Hypertension**	No	13,191 (51.71)	5295 (20.76)	7023 (27.53)	25,510 (81.88)
	Yes	1667 (29.51)	1346 (23.83)	2635 (46.66)	5,647 (18.12)
** Diabetes**	No	13,716 (50.72)	5686 (21.03)	7641 (28.25)	27,043 (86.80)
	Yes	1132 (27.52)	958 (23.29)	2023 (49.19)	4,113 (13.20)
** Heart disease**	No	14,175 (48.59)	6231 (21.36)	8765 (30.05)	29,172 (93.60)
	Yes	676 (33.88)	418 (20.95)	902 (45.16)	1,996 (6.40)
** Stroke**	No	14,358 (48.42)	6305 (21.26)	8989 (30.31)	29,652 (95.13)
	Yes	496 (32.66)	343 (22.59)	679 (44.75)	1,517 (4.87)
** Cancer**	No	14,333 (48.57)	6328 (21.44)	8848 (29.98)	29,508 (94.65)
	Yes	527 (31.58)	321 (19.27)	820 (49.15)	1,668 (5.35)
** Psychiatric disorder**	No	14,136 (47.81)	6341 (21.45)	9089 (30.74)	29,566 (94.90)
	Yes	710 (44.69)	305 (19.2)	574 (36.11)	1,590 (5.10)

*N* Un-weighted counts; percentages are weighted to account for the complex survey design and to make the estimates nationally representative, *BMI* Body mass index

Table 2 also indicated that in comparison to older adults with no high-risk waist circumference, the prevalence of single morbidity (20.10% Vs. 24.38%) and multimorbidity (25.29% Vs. 48.33%.) were higher among older adults with high-risk waist circumference. A similar pattern of single and multimorbidity prevalence was reported among older adults with a high-risk waist to hip ratio. It was further indicated that the multimorbidity prevalence was higher among older adults with the prevalence of both depressive symptoms and high-risk waist circumference (51.15%) and prevalence of both depressive symptoms and high-risk waist to hip ratio (41.01%) than any other combination group of depressive symptoms and high-risk waist circumference and depressive symptoms and high-risk waist to hip ratio respectively. The results also revealed that the prevalence of multimorbidity was higher among older adults with a family medical history of hypertension, diabetes, heart disease, stroke, cancer, and psychiatric disorder.

Table 3 presents multinomial logistic regression results of multimorbidity by key predictor variables. The table presents both crude odds and adjusted odds ratios for single morbidity and multimorbidity. Consistent with the crude odds ratio, adjusted models indicated that older adults with depressive symptoms had higher odds of single morbidity in comparison older adults with no depressive symptoms (AOR = 1.40, 95% CI: 1.17–1.68). In comparison to the normal BMI category, overweight and obese older adults were more likely to report single morbidity [(AOR = 1.62, 95% CI: 1.37–1.92 and (AOR = 2.14, 95% CI: 1.67–2.75), respectively]. Similarly, adjusted models indicated that older adults with depressive symptoms had higher odds of multimorbidity in comparison older adults with no depressive symptoms (AOR = 1.85, 95% CI: 1.58–2.16). In comparison to the normal BMI category, overweight and obese older adults were more likely to report multimorbidity [(AOR = 2.00, 95% CI: 1.72–2.33) and (AOR = 3.77, 95% CI: 2.94–4.82), respectively]. On the contrary, underweight older adults had lower odds of single and multimorbidity [(AOR = 0.85, 95% CI: 0.75–0.96) and (AOR = 0.66, 95% CI: 0.58–0.75) respectively] compared to older adults with normal BMI status. Further, in comparison to older adults with normal BMI status and no depressive symptoms, overweight older adults with depressive symptoms had higher odds of single and multimorbidity. Obese older adults with depressive symptoms had higher odds of multimorbidity. On the contrary, underweight older adults with no depressive symptoms had reported significantly lower odds of single and multimorbidity compared to older adults with normal BMI category and no depressive symptom [(AOR = 0.85, 95% CI: 0.75–0.96) and AOR = 0.67, 95% CI: 0.58–0.76), respectively].

**Table 3:** Crude and adjusted odds ratios from multinomial logistic regression of multimorbidity by key variables among older adults (Base category: No morbidity), Longitudinal Aging Study in India, 2017-19

**Variables**	**Categories**	**Crude OR (95% CI)**		**Adjusted OR (95% CI)**	
		**Single morbidity**	**Multimorbidity**	**Single morbidity**	**Multimorbidity**
**Depressive symptoms**	No	Ref.	Ref.	Ref.	Ref.
	Yes	1.22* (1.02–1.47)	1.61*** (1.38–1.89)	1.40*** (1.17–1.68)	1.85*** (1.58–2.16)
**BMI**	Normal	Ref.	Ref.	Ref.	Ref.
	Underweight	0.75*** (0.66–0.84)	0.53*** (0.47–0.60)	0.85** (0.75–0.96)	0.66*** (0.58–0.75)
	Overweight	2.01*** (1.69–2.38)	2.58*** (2.14–3.11)	1.62*** (1.37–1.92)	2.00*** (1.72–2.33)
	Obese	2.99*** (2.08–4.29)	5.36*** (3.55–8.09)	2.14*** (1.67–2.75)	3.77*** (2.94–4.82)
**High-risk waist circumference**	No	Ref.	Ref.	Ref.	Ref.
	Yes	2.43*** (2.11–2.80)	3.82*** (3.26–4.48)	1.88*** (1.64–2.16)	2.93*** (2.58–3.33)
**High-risk waist to hip ratio**	No	Ref.	Ref.	Ref.	Ref.
	Yes	1.51*** (1.34–1.70)	1.82*** (1.48–2.24)	1.25*** (1.11–1.42)	1.58*** (1.36–1.83)
**Depressive symptoms & BMI**	No & Normal	Ref.	Ref.	Ref.	Ref.
	No & Underweight	0.75*** (0.66–0.85)	0.53*** (0.47–0.60)	0.85* (0.75–0.96)	0.67*** (0.58–0.76)
	No & Overweight	2.04*** (1.71–2.44)	2.69*** (2.21–3.28)	1.63*** (1.37–1.95)	2.06*** (1.76–2.42)
	No & Obese	3.04*** (2.09–4.42)	5.44*** (3.52–8.41)	2.17*** (1.68–2.81)	3.82*** (2.95–4.93)
	Yes & Normal	1.33* (1.02–1.73)	1.87*** (1.51–2.32)	1.42** (1.09–1.84)	2.05*** (1.66–2.55)
	Yes & Underweight	0.92 (0.68–1.25)	0.89 (0.65–1.21)	1.13 (0.82–1.54)	1.11 (0.81–1.53)
	Yes & Overweight	2.12** (1.27–3.55)	3.15*** (1.99–5.01)	2.08** (1.30–3.34)	2.95*** (1.97–4.42)
	Yes & Obese	3.33* (1.24–8.95)	11.05*** (5.06–24.11)	2.66 (0.98–7.20)	8.13*** (3.34–19.79)
**Depressive symptoms & High-risk waist circumference**	No & No	Ref.	Ref.	Ref.	Ref.
	No & Yes	2.51*** (2.17–2.91)	4.02*** (3.40–4.75)	1.91*** (1.66–2.20)	3.00*** (2.62–3.43)
	Yes & No	1.29* (1.06–1.57)	1.78*** (1.49–2.12)	1.39** (1.14–1.70)	1.92*** (1.60–2.30)
	Yes & Yes	2.07** (1.25–3.42)	4.16*** (2.71–6.40)	2.18** (1.35–3.51)	4.67*** (3.19–6.82)
**Depressive symptoms & High-risk waist to hip ratio**	No & No	Ref.	Ref.	Ref.	Ref.
	No & Yes	1.55*** (1.37–1.76)	1.85*** (1.47–2.34)	1.28*** (1.12–1.47)	1.59*** (1.35–1.87)
	Yes & No	1.45* (1.07–1.97)	1.70** (1.19–2.43)	1.56** (1.13–2.17)	1.85*** (1.30–2.63)
	Yes & Yes	1.73*** (1.35–2.22)	2.82*** (2.11–3.78)	1.65*** (1.29–2.11)	2.89*** (2.29–3.63)
**Family medical history**					
** Hypertension**	No	Ref.	Ref.	Ref.	Ref.
	Yes	2.01*** (1.76–2.30)	2.97*** (2.50–3.53)	1.56*** (1.34–1.81)	1.81*** (1.58–2.07)
** Diabetes**	No	Ref.	Ref.	Ref.	Ref.
	Yes	2.04*** (1.75–2.38)	3.21*** (2.60–3.96)	1.24* (1.03–1.49)	1.45*** (1.24–1.70)
** Heart disease**	No	Ref.	Ref.	Ref.	Ref.
	Yes	1.41*** (1.15–1.72)	2.16*** (1.81–2.57)	1.04 (0.83–1.31)	1.21 (0.98–1.50)
** Stroke**	No	Ref.	Ref.	Ref.	Ref.
	Yes	1.57*** (1.26–1.97)	2.19*** (1.80–2.66)	1.32* (1.04–1.68)	1.52*** (1.22–1.88)
** Cancer**	No	Ref.	Ref.	Ref.	Ref.
	Yes	1.38* (1.02–1.87)	2.52*** (1.58–4.04)	0.90 (0.67–1.22)	1.29 (0.97–1.72)
** Psychiatric disorder**	No	Ref.	Ref.	Ref.	Ref.
	Yes	0.96 (0.79–1.17)	1.26* (1.02–1.55)	0.91 (0.73–1.13)	1.07 (0.85–1.36)

The control variables include age, sex, education, marital status, work status, physical activity, alcohol consumption, smoking, chewing tobacco, social participation, ill-treatment, wealth quintiles, religion, caste, place of residence and regions

*BMI* Body mass index

*if *p*-value < 0.05, ** if *p*-value < 0.005, *** if *p*-value < 0.001

In comparison to those with no high-risk waist circumference, older adults with a high risk of waist circumference had higher odds of single and multimorbidity [(AOR = 1.88, 95% CI: 1.64–2.16) and (AOR = 2.93, 95% CI: 2.58–3.33), respectively]. Similarly, it was found that older adults with a high-risk waist to hip ratio had higher odds of single and multimorbidity [(AOR = 1.25, 95% CI: 1.11–2.42) and (AOR = 1.58, 95% CI: 1.36–1.83), respectively] compared to older adults with no high-risk waist to hip ratio. The results also indicated that in comparison to older adults with no depressive symptom and no high-risk waist circumference or with no depressive symptom and no high-risk waist to hip ratio, having any or both of them significantly increased the odds of single and multimorbidity. In the adjusted model, having a family history of hypertension, diabetes, and stroke significantly increased the odds of single and multimorbidity. However, family history of heart disease, cancer, and psychiatric disorder did not significantly affect the prevalence of single and multimorbidity among older adults.

## Discussion

This cross-sectional study using a large country-representative data found significant associations between depressive symptoms, obesity, high-risk waist-hip ratio, waist circumference and multimorbidity among older adults in India. The association between depressive symptoms and multimorbidity was much stronger among those with adverse obesity-related measures than their healthy counterparts after adjusting for possible confounding factors.

Consistent with our findings, studies have shown that older adults with depressive symptoms tend to have unhealthier lifestyles, are less adherent with medication regimens, and have poorer self-care, which result in increased prevalence of chronic diseases [40, 41]. Multiple studies have also shown that chronic diseases can lead to chronic mental disorder among older adults and the possible mechanisms include the increased stress and anxiety among older patients [42–44], reduced care and support for the ill older persons from family members [45, 46], and the older individuals’ care burden and socioeconomic deprivation [47]. Similarly, evidence suggests that association between chronic diseases and mental disorders can be bidirectional. Just as the risk of suffering from comorbid diseases is increased by mental disorder, several chronic diseases alter mental physiology and lead to chronic somatic disease [48]. Although we used family history of psychiatric disorders as an instrumental variable of depressive symptoms and found no evidence of endogeneity in the models, future studies are required to determine the causal associations between our key variables.

Furthermore, our findings support the evidence that several measures of body composition including obesity, waist circumference and waist-hip ratio which are directly linked to increased body fat may lead to chronic diseases and multimorbidity in older individuals [39, 49–51]. Nevertheless, we found that underweight older adults had lower chances of multimorbidity in comparison to normal weight older adults. This is contrary to earlier evidence that underweight among older adults is associated with reduced functional ability, poorer quality of life, shorter life span and all-cause mortality compared with normal individuals [52–54]. This finding requires further investigation with longitudinal designs. Further, the study revealed a stronger combined effect of depressive symptoms and body composition on multimorbidity among older adults. Both general and abdominal obesity are associated with higher odds of multimorbidity among older adults in this study. A study conducted among the ageing population in China concluded that those with stable comorbidity, depression and obesity prevalence had an increased risk of functional disability [55]. Another study showed that the combined effect of obesity and depressive disorder on physical quality of life was larger than the sum of their separate effects [32]. Therefore, the possible risk of chronic conditions and chronic somatic disease might be addressed with a well-planned approach. A systematic review conducted on the association between obesity and depression showed that public health policy might target effectively controlling potential risk factors to improve population health. Such potential factors include socioeconomic status, health behaviour, psychosocial factors, and body composition [56].

Our findings further indicated that there is a strong effect of family history of hypertension, diabetes, and stroke on multimorbidity among older adults in India. Previous studies also revealed a clear association between the family history of hypertension, diabetes and stroke with their diagnoses [57–61]. Study showed that genetic factors or family history of diseases increase the risk of single and multimorbidity mainly by metabolic disease [62]. In the fully adjusted model, the results did not show any significant association between family medical history of heart disease and multimorbidity, however, evidence showed that cardiovascular diseases are likely to increase multimorbidity prevalence among ageing population [63]. A similar pattern was observed among population with a family history of cancer. A retrospective study conducted in Canada indicated that people with cancer are at high risk of at least one chronic disease [64]. However, the evidence for the association between a family history of cancer and their diagnosis is varied in the literature [21, 65, 66]. In general, an integrated healthcare approach is needed among older adults who are at higher risk of cancer to avoid poor health outcomes, which include identifying potential risk factors including family medical history of cancer [67]. Previous research established a strong association between psychiatric problems and the prevalence of morbidity [68–70] as well as multimorbidity among ageing population [71]. In the present study, family history of psychiatric disorders was not a significant predictor of morbidity in the fully adjusted model which need to be further investigated.

The main strength of the study is that the study utilised multiple anthropometric variables as potential risk factors of morbidity patterns with a nationally representative large sample data. We used measured data of anthropometric measures, which are considered more reliable than self-reported anthropometric data [72]. Similarly, the study used family history of diseases of the study participants, which is highly encouraged in public health policy practice, especially in case of difficulty of conducting genetic tests [73]. However, the study is not free from limitations. The present study is limited by its cross-sectional design, which limits the possibility of exploring directionality and causality among the hypothesised study variables. This issue could be addressed in the future by utilizing the forthcoming waves of LASI. At present, the study explored the association between selected variables with morbidity patterns. Studies established that the prevalence of morbidity might be potentially contributed by several factors such as healthcare access and utilisation [74]. The study does not consider such dimensions. Future studies could also be extended to synthesise the role of multimorbidity as a potential factor for functional decline, healthcare expenditure, poor quality of life and wellbeing, hospitalization, and mortality.

## Conclusions

The findings revealed that the presence of depressive symptoms, overweight or obesity, and high-risk anthropometric measures such as high-risk waist circumference and high-risk waist to hip ratio increased the risk of morbidity among older adults in India. The findings further indicated a higher risk of morbidity among the older population, especially those with a family history of hypertension, diabetes, and stroke. It is suggested to adopt an integrated public health policy approach to control depressive symptoms and high-risk body composition factors to strategically prepare against the elevated risk of morbidity among ageing population.

## Data Availability

The data are publicly available at the Gateway to Global Aging Data (https://g2aging.org/ ).

## References

[1] WHO. WHO Factsheet on depressive disorder. Depressive disorder (depression). 2021. Available from: https://www.who.int/news-room/fact-sheets/detail/depression. Cited 2023 Aug 20.

[2] Sagar R, Dandona R, Gururaj G, Dhaliwal RS, Singh A, Ferrari A (2020). The burden of mental disorders across the States of India: the global burden of Disease Study 1990–2017. Lancet Psychiatry.

[3] Triolo F, Sjöberg L, Calderón-Larrañaga A, Belvederi Murri M, Vetrano DL, Fratiglioni L (2023). Late-life depression and multimorbidity trajectories: the role of symptom complexity and severity. Age Ageing.

[4] Hildrum B, Mykletun A, Holmen J, Dahl AA (2008). Effect of anxiety and depression on blood pressure: 11-Year longitudinal population study. Br J Psychiatry.

[5] Everson SA, Kaplan GA, Goldberg DE, Salonen JT (2000). Hypertension incidence is predicted by high levels of hopelessness in Finnish men. Hypertension.

[6] Black S, Markides K, Ray L (2003). Depression predicts increased incidence of adverse Health outcomes in older Mexican americans with type 2 diabetes. Diabetes Care J.

[7] Obesity and overweight. WHO; 2021. Available from: https://www.who.int/news-room/fact-sheets/detail/obesity-and-overweight. Cited 2022 Oct 19.

[8] Dai H, Alsalhe TA, Chalghaf N, Riccò M, Bragazzi NL, Wu J (2020). The global burden of disease attributable to high body mass index in 195 countries and territories, 1990–2017: an analysis of the global burden of Disease Study. PLoS Med.

[9] Lee HA, Park H (2021). Comorbidity network analysis related to obesity in middle-aged and older adults: findings from Korean population-based survey data. Epidemiol Health.

[10] Kivimäki M, Strandberg T, Pentti J, Nyberg ST, Frank P, Jokela M (2022). Body-mass index and risk of obesity-related complex multimorbidity: an observational multicohort study. Lancet Diabetes Endocrinol.

[11] Haregu TN, Lee JT, Oldenburg B, Armstrong G (2020). Comorbid depression and obesity: correlates and synergistic association with noncommunicable diseases among Australian men. Prev Chronic Dis.

[12] Mannan M, Mamun A, Doi S, Clavarino A (2016). Is there a bi-directional relationship between depression and obesity among adult men and women? Systematic review and bias-adjusted meta analysis. Asian J Psychiatry.

[13] Faith MS, Butryn M, Wadden TA, Fabricatore A, Nguyen AM, Heymsfield SB (2011). Evidence for prospective associations among depression and obesity in population-based studies: prospective obesity-depression associations. Obes Rev.

[14] Pedditizi E, Peters R, Beckett N (2016). The risk of overweight/obesity in mid-life and late life for the development of dementia: a systematic review and meta-analysis of longitudinal studies. Age Ageing.

[15] Luppino FS, de Wit LM, Bouvy PF, Stijnen T, Cuijpers P, Penninx BWJH (2010). Overweight, obesity, and depression: a systematic review and meta-analysis of longitudinal studies. Arch Gen Psychiatry.

[16] Sikorski C, Luppa M, Luck T, Riedel-Heller SG (2015). Weight stigma gets under the skin—evidence for an adapted psychological mediation framework—a systematic review. Obesity.

[17] Mozaffarian D, Benjamin EJ, Go AS, Arnett DK, Blaha MJ, Cushman M et al. Heart disease and stroke statistics—2016 update: a report from the American Heart Association. Circulation. 2016;133(4). Available from: https://www.ahajournals.org/doi/10.1161/CIR.0000000000000350. Cited 2022 Oct 19. 10.1161/CIR.0000000000000350 26673558

[18] Kim H, Friedlander Y, Longstreth WT, Edwards KL, Schwartz SM, Siscovick DS (2004). Family history as a risk factor for stroke in young women. Am J Prev Med.

[19] Valdez R, Yoon PW, Liu T, Khoury MJ (2007). Family history and prevalence of diabetes in the US population: the 6-year results from the National Health and Nutrition Examination Survey (1999–2004). Diabetes Care.

[20] Ranasinghe P, Cooray DN, Jayawardena R, Katulanda P (2015). The influence of family history of hypertension on disease prevalence and associated metabolic risk factors among Sri Lankan adults. BMC Public Health.

[21] Ramsey SD, Yoon P, Moonesinghe R, Khoury MJ (2006). Population-based study of the prevalence of family history of cancer: implications for cancer screening and prevention. Genet Med.

[22] Clark AM, DesMeules M, Luo W, Duncan AS, Wielgosz A (2009). Socioeconomic status and cardiovascular disease: risks and implications for care. Nat Rev Cardiol.

[23] de Mestral C, Stringhini S (2017). Socioeconomic status and cardiovascular disease: an update. Curr Cardiol Rep.

[24] Rooks RN, Simonsick EM, Miles T, Newman A, Kritchevsky SB, Schulz R (2002). The association of race and socioeconomic status with cardiovascular disease indicators among older adults in the health, aging, and body composition study. J Gerontol - Ser B Psychol Sci Soc Sci.

[25] Subramanian SV, Corsi DJ, Subramanyam MA, Smith GD (2013). Jumping the gun: the problematic discourse on socioeconomic status and cardiovascular health in India. Int J Epidemiol.

[26] Srivastava S, Muhammad T (2020). Violence and associated health outcomes among older adults in India: a gendered perspective. SSM-Popul Health.

[27] Wong JS, Waite LJ (2017). Elder mistreatment predicts later physical and psychological health: results from a national longitudinal study. J Elder Abuse Negl.

[28] Emberson JR, Bennett DA (2006). Effect of alcohol on risk of coronary heart disease and stroke: causality, bias, or a bit of both?. Vasc Health Risk Manag.

[29] Shield KD, Parry C, Rehm J (2013). Chronic diseases and conditions related to Alcohol Use. Alcohol Res Curr Rev.

[30] Al-Bashaireh AM, Haddad LG, Weaver M, Kelly DL, Chengguo X, Yoon S (2018). The effect of tobacco smoking on musculoskeletal health: a systematic review. J Environ Public Health.

[31] Mishra VK, Srivastava S, Muhammad T, Murthy PV (2022). Relationship between tobacco use, alcohol consumption and non-communicable diseases among women in India: evidence from National Family Health Survey-2015-16. BMC Public Health.

[32] Nigatu YT, Reijneveld SA, de Jonge P, van Rossum E, Bültmann U (2016). The combined effects of obesity, abdominal obesity and major depression/anxiety on health-related quality of life: the lifelines cohort study. PLoS ONE.

[33] International Institute for Population Sciences (IIPS) N MoHFW, Harvard TH Chan School of Public Health (HSPH) and the University of Southern California (USC). Longitudinal Ageing Study in India (LASI) Wave 1, 2017-18, India Report. Mumbai; 2020. Available from: https://www.iipsindia.ac.in/lasi/.

[34] Meher T, Muhammad T, Gharge S. The Association between single and multiple chronic conditions and depression among older Population in India: a comparative study between men and women. Int J Geriatr Psychiatry. 2021;37(1). 10.1002/gps.5639 34633709

[35] Muhammad T, Meher T, Sekher TV (2021). Association of elder abuse, crime victimhood and perceived neighbourhood safety with major depression among older adults in India: a cross-sectional study using data from the LASI baseline survey (2017–2018). BMJ Open.

[36] Kessler RC, Üstün BB (2004). The World Mental Health (WMH) Survey Initiative version of the World Health Organization (WHO) Composite International Diagnostic interview (CIDI). Int J Methods Psychiatr Res.

[37] Trainor K, Mallett J, Rushe T (2013). Age related differences in mental health scale scores and depression diagnosis: adult responses to the CIDI-SF and MHI-5. J Affect Disord.

[38] Haro JM, Arbabzadeh-Bouchez S, Brugha TS, Girolamo G, De, Guyer ME, Jin R (2008). Concordance of the Composite International Diagnostic interview Version 3.0 (CIDI 3.0) with standardized clinical assessments in the WHO World Mental Health Surveys. Int J Methods Psychiatr Res.

[39] Zhang J, Xu L, Li J, Sun L, Qin W (2020). Association between obesity-related anthropometric indices and multimorbidity among older adults in Shandong, China: a cross-sectional study. BMJ Open.

[40] Agustini B, Lotfaliany M, Woods RL, McNeil JJ, Nelson MR, Shah RC (2020). Patterns of Association between depressive symptoms and Chronic Medical morbidities in older adults. J Am Geriatr Soc.

[41] Almeida OP, Alfonso H, Pirkis J, Kerse N, Sim M, Flicker L (2011). A practical approach to assess depression risk and to guide risk reduction strategies in later life. Int Psychogeriatr.

[42] Chapman DP, Perry GS, Strine TW (2005). Peer reviewed: the vital link between chronic disease and depressive disorders. Prev Chronic Dis.

[43] Cohen BE, Edmondson D, Kronish IM (2015). State of the art review: depression, stress, anxiety, and cardiovascular disease. Am J Hypertens.

[44] Jiang CH, Zhu F, Qin TT (2020). Relationships between chronic diseases and depression among middle-aged and elderly people in China: a prospective study from CHARLS. Curr Med Sci.

[45] Golics CJ, Basra MKA, Finlay AY, Salek S (2013). The impact of disease on family members: a critical aspect of medical care. J R Soc Med.

[46] Larkin J, Foley L, Smith SM, Harrington P, Clyne B (2021). The experience of financial burden for people with multimorbidity: a systematic review of qualitative research. Health Expect.

[47] Gonzalez-Gonzalez AI, Brünn R, Nothacker J, Dinh TS, Brueckle MS, Dieckelmann M (2021). Everyday lives of middle-aged persons living with multimorbidity: protocol of a mixed-methods systematic review. BMJ Open.

[48] Snoek FJ, Bremmer MA, Hermanns N (2015). Constructs of depression and distress in diabetes: time for an appraisal. Lancet Diabetes Endocrinol.

[49] Marengoni A, Angleman S, Melis R, Mangialasche F, Karp A, Garmen A (2011). Aging with multimorbidity: a systematic review of the literature. Ageing Res Rev.

[50] Muhammad T, Boro B, Kumar M, Srivastava S (2022). Gender differences in the association of obesity-related measures with multi-morbidity among older adults in India: evidence from LASI, Wave-1. BMC Geriatr.

[51] Srivastava S, Kj VJ, Dristhi D, Muhammad T (2021). Interaction of physical activity on the association of obesity-related measures with multimorbidity among older adults: a population-based cross-sectional study in India. BMJ Open.

[52] Amankwaa I, Nelson K, Rook H, Hales C (2022). Association between body mass index, multi-morbidity and activities of daily living among New Zealand nursing home older adults: a retrospective analysis of nationwide InterRAI data. BMC Geriatr.

[53] Minagawa Y, Saito Y (2021). The role of underweight in active life expectancy among older adults in Japan. J Gerontol Ser B.

[54] Kim HJ, Kim BS, Lee JH, Shin JH (2022). Impact of underweight on 3-year all-cause mortality in patients with acute severe hypertension: a retrospective cohort study. Sci Rep.

[55] Lin L, Bai S, Qin K, King C, Wong H, Wu T, et al. Comorbid depression and obesity, and its transition on the risk of functional disability among middle – aged and older Chinese : a cohort study. BMC Geriatrics. 2022;22(1):1–10. 10.1186/s12877-022-02972-1 PMC8976974 35366819

[56] Preiss K, Brennan L, Clarke D (2013). A systematic review of variables associated with the relationship between obesity and depression. Obes Rev.

[57] Sande MAB, Van Der, Walraven GEL, Milligan PJM, Banya WAS, Ceesay SM, Nyan OA (2001). Family history: an opportunity for early interventions and improved control of hypertension, obesity and diabetes. Bull World Health Organ.

[58] Meneilly GS, Tessier D (2001). Diabetes in Elderly Adults. J Gerontol A Biol Sci Med Sc.

[59] Liu M, He Y, Jiang B, Wang J, Wu L, Wang Y (2015). Association between family history and hypertension among Chinese elderly. Med (Baltim).

[60] Pourasgari M, Mohamadkhani A (2020). Heritability for stroke: essential for taking family history. Casp J Intern Med.

[61] Muhammad T, Irshad CV, Rajan SI (2022). BMI mediates the association of family medical history with self-reported hypertension and diabetes among older adults: evidence from baseline wave of the longitudinal aging study in India. SSM-Popul Health.

[62] Lu J, Wang Y, Hou L, Zuo Z, Zhang N, Wei A. Multimorbidity patterns in old adults and their associated multi-layered factors: a cross-sectional study. BMC Geriatrics. 2021;21(1):372. 10.1186/s12877-021-02292-w PMC8214251 34147073

[63] Dunlay SM, Chamberlain AM (2016). Multimorbidity in older patients with Cardiovascular Disease. Curr Cardiovasc Risk Rep.

[64] Koné AP, Scharf D (2021). Prevalence of multimorbidity in adults with cancer, and associated health service utilization in Ontario, Canada : a population- based retrospective cohort study. BMC Cancer.

[65] Murff HJ, Peterson NB, Greevy R, Zheng W (2006). Impact of patient age on family cancer history. Genet Med.

[66] Kharazmi E, Fallah M, Sundquist K, Hemminki K (2012). Familial risk of early and late onset cancer: nationwide prospective study. BMJ.

[67] Guntupalli AM, Selvamani Y, Maclennan SJ, Dilip TR. Health status and associated factors of middle – aged and older adult cancer survivors in India : results from the longitudinal ageing study in India. BMC Cancer. 2022;2(1):1–12 10.1186/s12885-022-10111-7 PMC9587652 36273166

[68] Melis R, Marengoni A, Angleman S, Fratiglioni L (2014). Incidence and predictors of multimorbidity in the elderly: a population-based longitudinal study. PLoS ONE.

[69] Pati S, Mahapatra P, Dwivedi R, Athe R, Sahoo KC, Samal M (2021). Multimorbidity and its outcomes among patients attending Psychiatric Care settings: an observational study from Odisha. India. Front Public Health.

[70] Singh S, Shri N, Dwivedi LK (2022). An association between multi-morbidity and depressive symptoms among Indian adults based on propensity score matching. Sci Rep.

[71] Chireh B, Essien SK, Novik N (2021). Multimorbidity, disability, and mental health conditions in a nationally representative sample of middle-aged and older canadians. J Affect Disord Rep.

[72] Aminianfar A, Saneei P, Nouri M, Shafiei R, Hassanzadeh-Keshteli A, Esmaillzadeh A (2021). Validity of self-reported height, weight, body Mass Index, and Waist circumference in Iranian adults. Int J Prev Med.

[73] Yoon PW, Scheuner MT, Peterson-Oehlke KL, Gwinn M, Faucett A, Khoury MJ (2002). Can family history be used as a tool for public health and preventive medicine?. Genet Med.

[74] Balakrishnan S, Karmacharya I, Ghimire S, Mistry SK, Singh DR, Yadav OP (2022). Prevalence of multimorbidity and its correlates among older adults in Eastern Nepal. BMC Geriatr.

